# Experimental data for physical characteristics, fiber compositions, and tensile properties of nonwoven wipes and toilet papers

**DOI:** 10.1016/j.dib.2019.104818

**Published:** 2019-11-16

**Authors:** Serkan Durukan, Fatih Karadagli

**Affiliations:** Department of Environmental Engineering, School of Engineering, Sakarya University, Esentepe, Sakarya, 54187, Turkey

**Keywords:** Physical characteristics, Fiber compositions, Tensile properties, Nonwoven wipes, Toilet papers

## Abstract

This article presents experimental data for physical characteristics, fiber compositions, and tensile properties of non-flushable wipes, flushable wipes, and toilet papers. Samples included 42 flushable wipes, 16 non-flushable wipes, and 11 toilet papers that were collected from around the world by considering product diversity in their retail regions (e.g., north america, and europe), manufacturers (e.g., global, and regional), and function (e.g., baby, toddler, patient, adult, and feminine wipes). The data were generated in accordance with relevant standard methods of International Organization for Standardization (ISO). The data are provided here in full (not hosted by any public repository) in association with the research article: “Physical characteristics, fiber compositions, and tensile properties of nonwoven wipes and toilet papers in relevance to what is flushable” [1]. Readers are referred to the research article for discussions and interpretations of the data presented in this document.

**Table:** Specifications Table

Subject area	Environmental Engineering
More specific subject area	Wastewater collection and treatment
Type of data	Tables and images
How data was acquired	Leica VMHT MOT microscope (Leica Microsystems GmbH, Wetzlar, Germany) was operated at 100X magnification to quantify sheet thicknesses of samples. Olympus BX52 microscope (Olympus Corp., Tokyo, Japan) that was equipped with a digital camera was operated at 100X magnification to capture microscopic images of fiber types. Schimadzu autograph AG-IC series (Schimadzu Corp., Tokyo, Japan) was used to quantify tensile properties of samples. The testing instrument was operated by an experienced staff using a personal computer and Trapezium X software (Schimadzu Corp., Tokyo, Japan), which served as a specific interface between operator and testing instrument.
Data format	Raw, and analyzed
Experimental factors	For measurements in dry-states of samples, moist sheets (i.e., as in their retail package) were dried at 40 °C for 24 h.
Experimental features	Physical characteristics of samples include width, length, surface area, sheet thickness, sheet volume, sheet mass, basis weight, specific volume, and moisture content. Fiber compositions were identified by using Dupont Stain No.4, and Herzberg stain. Tensile properties include the maximum force required to break a specimen (F_max_), tensile strength, tensile index, breaking length, and elongation at break.
Data source location	Sakarya University, Faculty of Engineering, Department of Environmental Engineering, Esentepe Campus, Serdivan, Sakarya, Turkey, 54187
Data accessibility	Data are with this article
Related research article	Durukan, S. and Karadagli, F. 2019. Physical characteristics, fiber compositions, and tensile properties of nonwoven wipes and toiler papers in relevance to what is flushable. Science of the Total Environment, 697, 134135, DOI: https://doi.org/10.1016/j.scitotenv.2019.134135

**Table:** 

**Value of the Data** • The data elucidate whether or not flushable wipes are similar to non-flushable wipes, or to toilet papers, based on their physical characteristics, fiber compositions, and tensile properties. • The data can be used to design new studies to assess how sanitary consumer products (flushable, or non-flushable) will move and disintegrate in wastewater collection and treatment systems. • The data are beneficial to relevant product manufacturers to improve existing products, or to design new ones that will meet consumer expectations and will be compatible with wastewater operations. • The data can serve as a technical basis for development of standards and regulations to specify sanitary products that will be disposed of via wastewater collection systems.

## 1 Data

### 1.1 Physical characteristics

Table 1, Table 2 present physical characteristics of non-flushable wipes in their moist-as-received states, and in their dry states, respectively. Table 3, Table 4, Table 5, Table 6 present the same information for flushable wipe samples. Essential statistics of the data are available in the last two rows of relevant tables. Interpretations and discussions of the data are provided in our associated article [1]. For physical characteristics of toilet papers, we refer the readers to our previous publication [2].

**Table 1:** Physical characteristics of non-flushable moist wipe samples from around the world. Physical characteristics were quantified by using moist sheets in their as-received state (e.g., as in their retail package). Sample IDs indicate NF: Non-flushable wipe, and SN: Sample Number.

No.	I.D.	Sheet Mass (g/sheet)	Surface Area (Length x Width) (cm^2^)	Sheet Thickness (μm)	Sheet Volume (cm^3^)	Basis Weight (g/m^2^)	Specific Volume (dm^3^/kg)
1	NF-SN-1	6.0	340	336	11.4	176	1.9
2	NF-SN-2	6.5	302	342	10.3	215	1.6
3	NF-SN-3	7.2	349	321	11.2	207	1.5
4	NF-SN-4	5.1	371	330	12.2	137	2.2
5	NF-SN-5	5.2	312	312	9.8	166	1.8
6	NF-SN-6	4.1	334	300	10.4	124	2.4
7	NF-SN-7	3.6	328	315	9.5	108	2.9
8	NF-SN-8	3.2	332	285	9.9	95	2.8
9	NF-SN-9	3.9	294	335	9.8	134	2.5
10	NF-SN-10	3.6	213	330	7.0	168	1.9
11	NF-SN-11	6.9	353	339	12.0	196	1.9
12	NF-SN-12	6.5	362	376	13.6	191	2.0
13	NF-SN-13	4.8	282	236	6.7	232	1.4
14	NF-SN-14	2.6	231	306	7.0	205	3.4
15	NF-SN-15	4.5	274	213	5.8	94	1.4
16	NF-SN-16	4.9	296	335	10.0	152	2.2
	**Average**	**4.9**	**311**	**313**	**9.8**	**163**	**2.1**
	**Range**	**2.6**–**7.2**	**213**–**371**	**213**–**376**	**5.8**–**13.6**	**94**–**232**	**1.4–3.4**
	***s***∗	**1.4**	**45**	**40**	**2.2**	**44**	**0.6**
	**ε**∗	**0.4**	**11**	**10**	**0.6**	**11**	**0.14**

∗ “*s*” means standard deviation, and “ε” means standard error.

**Table 2:** Physical characteristics of dry non-flushable wipe samples from around the world. Physical characteristics were quantified by using sheets that were dried at 40 °C for 24 h. Sample IDs indicate NF: Non-flushable wipe, and SN: Sample Number.

No.	I.D.	Sheet Mass (g/sheet)	Surface Area (Length x Width) (cm^2^)	Sheet Thickness (μm)	Sheet Volume (cm^3^)	Basis Weight (g/m^2^)	Specific Volume (dm^3^/kg)	Moisture (%)
1	NF-SN-1	1.8	347	448	15.5	52	8.6	70
2	NF-SN-2	1.5	281	531	14.9	55	9.7	76
3	NF-SN-3	1.9	324	351	11.4	59	6.0	74
4	NF-SN-4	1.4	300	336	10.1	48	7.1	72
5	NF-SN-5	1.4	291	419	12.2	47	8.8	73
6	NF-SN-6	1.4	323	283	9.1	44	6.4	66
7	NF-SN-7	1.3	319	434	13.9	40	10.8	64
8	NF-SN-8	1.2	315	493	15.5	38	12.8	62
9	NF-SN-9	1.2	283	312	8.9	43	7.3	69
10	NF-SN-10	1.2	203	288	5.8	58	5.0	67
11	NF-SN-11	1.9	320	416	13.3	59	7.1	71
12	NF-SN-12	1.8	333	386	12.8	55	7.0	72
13	NF-SN-13	1.4	266	352	9.4	51	6.9	72
14	NF-SN-14	1.0	273	390	10.6	46	8.4	61
15	NF-SN-15	1.6	280	178	4.9	52	3.4	58
16	NF-SN-16	1.2	296	323	9.6	77	4.2	71
	**Average**	**1.5**	**297**	**371**	**11.1**	**52**	**7.5**	**69**
	**Range**	**1.0**–**1.9**	**203**–**347**	**178**–**531**	**5.0**–**15.5**	**38**–**77**	**3.4**–**12.8**	**58**–**76**
	***s***∗	**0.3**	**34**	**87**	**3.2**	**8**	**2.4**	**5**
	**ε**∗	**0.07**	**9**	**22**	**0.8**	**2**	**0.6**	**1**

∗ “*s*” means standard deviation, and “ε” means standard error.

**Table 3:** Physical characteristics of flushable moist wipe samples from North America. Physical characteristics were quantified by using moist sheets in their as-received state (e.g., as in their retail package). Sample IDs indicate NA: North America (flushable), and SN: Sample Number.

No.	I.D.	Sheet Mass (g/sheet)	Surface Area (Length x Width) (cm^2^)	Sheet Thickness (μm)	Sheet Volume (cm^3^)	Basis Weight (g/m^2^)	Specific Volume (dm^3^/kg)
1	NA-SN-1	3.8	180	320	5.7	210	1.5
2	NA-SN-2	5.5	250	327	8.2	220	1.5
3	NA-SN-3	4.2	208	311	6.5	202	1.5
4	NA-SN-4	4.9	237	305	7.2	206	1.5
5	NA-SN-5	5.2	237	343	8.2	218	1.6
6	NA-SN-6	4.6	236	316	7.5	194	1.6
7	NA-SN-7	4.0	237	276	6.5	170	1.6
8	NA-SN-8	5.0	241	297	7.2	209	1.4
9	NA-SN-9	5.8	274	227	6.2	212	1.1
10	NA-SN-10	6.3	203	413	8.4	311	1.3
11	NA-SN-11	4.5	261	391	10.2	171	2.3
12	NA-SN-12	4.3	213	319	6.8	203	1.6
13	NA-SN-13	5.0	232	412	9.5	216	1.9
14	NA-SN-14	4.1	250	381	9.6	164	2.3
15	NA-SN-15	3.7	201	373	7.5	186	2.0
16	NA-SN-16	3.8	269	343	9.2	140	2.5
	**Average**	**4.7**	**233**	**335**	**7.8**	**202**	**1.7**
	**Range**	**3.7**–**6.3**	**180**–**274**	**227**–**413**	**5.7**–**10.2**	**140**–**311**	**1.1**–**2.5**
	***s***∗	**0.8**	**26**	**50**	**1.3**	**37**	**0.4**
	**ε**∗	**0.2**	**7**	**13**	**0.3**	**9**	**0.1**

∗ “*s*” means standard deviation, and “ε” means standard error.

**Table 4:** Physical characteristics of dry flushable wipe samples from North America. Physical characteristics were quantified by using sheets that were dried at 40 °C for 24 h. Sample IDs indicate NA: North America (flushable), and SN: Sample Number.

No.	I.D.	Sheet Mass (g/sheet)	Surface Area (Length x Width) (cm^2^)	Sheet Thickness (μm)	Sheet Volume (cm^3^)	Basis Weight (g/m^2^)	Specific Volume (dm^3^/kg)	Moisture (%)
1	NA-SN-1	1.1	170	327	5.6	62	5.3	72
2	NA-SN-2	1.5	237	263	6.2	64	4.1	73
3	NA-SN-3	1.5	190	572	10.9	80	7.2	64
4	NA-SN-4	1.8	222	631	14.0	81	7.8	63
5	NA-SN-5	1.7	220	565	12.4	79	7.2	66
6	NA-SN-6	1.6	222	449	9.9	74	6.1	64
7	NA-SN-7	1.3	219	309	6.8	59	5.2	68
8	NA-SN-8	1.6	237	264	6.2	67	3.9	68
9	NA-SN-9	1.8	260	317	8.3	67	4.7	70
10	NA-SN-10	1.4	197	407	8.0	70	5.8	78
11	NA-SN-11	1.4	255	296	7.6	53	5.6	70
12	NA-SN-12	1.6	203	437	8.9	79	5.5	63
13	NA-SN-13	1.4	226	359	8.1	61	5.9	72
14	NA-SN-14	1.4	250	330	8.2	55	6.0	61
15	NA-SN-15	1.2	200	314	6.3	58	5.4	58
16	NA-SN-16	1.3	261	420	11.0	51	8.2	71
	**Average**	**1.5**	**223**	**391**	**8.6**	**66**	**5.9**	**68**
	**Range**	**1.1**–**1.8**	**170**–**261**	**263**–**631**	**5.6**–**14**	**51**–**81**	**3.9–8.2**	**58**–**78**
	***s***∗	**0.2**	**27**	**114**	**2.4**	**10**	**1.2**	**5**
	**ε**∗	**0.05**	**7**	**29**	**0.6**	**2**	**0.3**	**1.3**

∗ “*s*” means standard deviation, and “ε” means standard error.

**Table 5:** Physical characteristics of flushable wipe samples from European, and from Far Eastern countries. Physical characteristics were quantified by using moist sheets in their as-received state (e.g., as in their retail package). Sample IDs indicate EU: Europe (flushable), FE: Far East (flushable), and SN: Sample Number.

No.	I.D.	Sheet Mass (g/sheet)	Surface Area (Length x Width) (cm^2^)	Sheet Thickness (μm)	Sheet Volume (cm^3^)	Basis Weight (g/m^2^)	Specific Volume (dm^3^/kg)
1	EU-SN-1	4.3	255	239	6.1	168	1.4
2	EU-SN-2	4.3	267	303	8.1	160	1.9
3	EU-SN-3	5.1	228	292	6.7	222	1.3
4	EU-SN-4	4.6	236	387	9.1	196	2.0
5	EU-SN-5	4.7	235	397	9.3	201	2.0
6	EU-SN-6	2.9	205	185	3.8	141	1.3
7	EU-SN-7	2.9	244	320	7.8	117	2.7
8	EU-SN-8	4.6	227	335	7.6	204	1.6
9	EU-SN-9	4.9	252	335	8.4	195	1.7
10	EU-SN-10	3.9	223	150	3.3	177	0.8
11	EU-SN-11	4.5	200	337	6.7	227	1.5
12	EU-SN-12	4.5	269	266	7.2	167	1.6
13	EU-SN-13	4.2	264	238	6.3	159	1.5
14	EU-SN-14	3.9	222	255	5.7	178	1.4
15	EU-SN-15	3.8	213	326	6.9	177	1.8
16	EU-SN-16	5.2	222	321	7.1	233	1.4
17	EU-SN-17	5.2	222	318	7.1	234	1.4
18	EU-SN-18	5.0	206	352	7.3	244	1.4
19	FE-SN-1	4.0	306	314	9.6	127	2.4
20	FE-SN-2	4.3	385	382	14.7	111	3.4
21	FE-SN-3	6.0	338	421	14.6	177	2.4
22	FE-SN-4	5.8	374	364	15.7	155	2.7
23	FE-SN-5	4.3	270	312	8.4	159	2.0
24	FE-SN-6	6.8	446	297	12.9	152	1.9
25	FE-SN-7	3.8	366	185	6.6	103	1.7
26	FE-SN-8	5.3	389	248	9.8	136	1.9
	**Average**	**4.6**	**272**	**303**	**8.3**	**174**	**1.8**
	**Range**	**2.9**–**6.8**	**200**–**446**	**150**–**421**	**3.3**–**15.7**	**103**–**244**	**0.8**–**3.4**
	***s***∗	**0.9**	**68**	**67**	**3**	**38**	**0.5**
	**ε**∗	**0.2**	**13**	**13**	**0.6**	**8**	**0.1**

∗ “*s*” means standard deviation, and “ε” means standard error.

**Table 6:** Physical characteristics of flushable wipe samples from European, and from Far Eastern Countries. The characteristics were quantified by using sheets that were dried at 40 °C for 24 h. Sample IDs indicate EU: Europe (flushable), FE: Far East (flushable), and SN: Sample Number.

No.	I.D.	Sheet Mass (g/sheet)	Surface Area (Length x Width) (cm^2^)	Sheet Thickness (μm)	Sheet Volume (cm^3^)	Basis Weight (g/m^2^)	Specific Volume (dm^3^/kg)	Moisture (%)
1	EU-SN-1	1.4	244	273	6.6	59	4.6	66
2	EU-SN-2	1.4	254	311	7.9	55	5.7	68
3	EU-SN-3	1.3	214	310	6.6	63	4.9	73
4	EU-SN-4	1.5	228	287	6.5	65	4.4	68
5	EU-SN-5	1.5	224	307	6.9	65	4.7	69
6	EU-SN-6	1.0	197	246	4.8	52	4.7	65
7	EU-SN-7	1.1	237	174	4.1	48	3.7	61
8	EU-SN-8	1.4	231	298	6.9	63	4.8	69
9	EU-SN-9	1.6	237	356	8.4	68	5.3	76
10	EU-SN-10	1.1	200	221	4.4	54	4.1	67
11	EU-SN-11	1.3	195	305	6.0	69	4.5	66
12	EU-SN-12	1.5	252	351	8.8	60	5.8	67
13	EU-SN-13	1.5	247	190	4.7	59	3.2	75
14	EU-SN-14	1.1	202	352	7.1	52	6.8	73
15	EU-SN-15	1.1	203	340	6.9	56	6.0	70
16	EU-SN-16	1.4	228	310	7.0	60	5.2	74
17	EU-SN-17	1.4	222	378	8.4	64	5.9	73
18	EU-SN-18	1.4	206	363	7.5	67	5.4	73
19	FE-SN-1	1.2	289	313	8.9	42	7.4	70
20	FE-SN-2	1.4	357	294	10.4	39	7.4	67
21	FE-SN-3	2.3	344	545	18.6	67	8.0	62
22	FE-SN-4	2.0	343	532	18.2	58	9.1	65
23	FE-SN-5	1.2	273	482	13.1	44	10.9	72
24	FE-SN-6	2.3	433	510	22.1	53	9.6	66
25	FE-SN-7	1.6	356	234	8.2	45	5.1	58
26	FE-SN-8	2.0	376	531	20	53	10.0	62
	**Average**	**1.5**	**261**	**339**	**9.2**	**57**	**6.0**	**68**
	**Range**	**1.0**–**2.3**	**195**–**433**	**174**–**545**	**4.1**–**22**	**39**–**69**	**3.2**–**10.9**	**58**–**76**
	***s***∗	**0.35**	**66**	**104**	**5**	**8**	**2**	**5**
	**ε**∗	**0.07**	**13**	**20**	**1**	**2**	**0.4**	**1**

∗ “*s*” means standard deviation, and “ε” means standard error.

Our data are composed of raw, and analyzed quantities as follows: 1- A physical property, such as sheet mass, was quantified by measuring sheet masses of several specimens of a sample; then, the arithmetic average of these measured values was reported as the sheet mass of that sample, 2- A physical property, such as surface area, was computed as the product of length and width of a sheet. For clarity, we use an example case (sample no.1 in Table 1) to demonstrate step-by-step how we quantified the data for that sample.

For sheet mass measurements, we removed three separate sheets from top, middle, and bottom parts of a sample's package. Then, we measured mass of each sheet gravimetrically, estimated arithmetic average of the three measurements, and reported this value as the sheet mass of that sample. We present below the individual readings, the average sheet mass, and the reported value for sample no.1 in Table 1 as an example case.

**Table:** 

Sheet mass measurement-1 (g)	Sheet mass measurement-2 (g)	Sheet mass measurement-3 (g)	Arithmetic average of sheet mass measurements (g)	Reported sheet mass value (g)
5.97	6.04	6.01	6.00	6.0

For length, and width of a sheet, we used two separate sheets to quantify each dimension, and then, we estimated surface area as the product of length and width. For the example case (sample no.1 in Table 1), our measurements, the estimated surface areas, and the arithmetic average of surface areas were quantified as follows

**Table:** 

Length measurement-1 (cm)	Width measurement-1 (cm)	Surface area-1 (length x width) (cm^2^)	Average of surface areas (cm^2^)	Reported surface area (cm^2^)
20.4	16.7	340.7	(340.7 + 338.7)/2 = 340	340
Length measurement-2 (cm)	Width measurement-2 (cm)	Surface area-2 (length x width) (cm^2^)		
20.4	16.6	338.7		

**Fig. 1:**
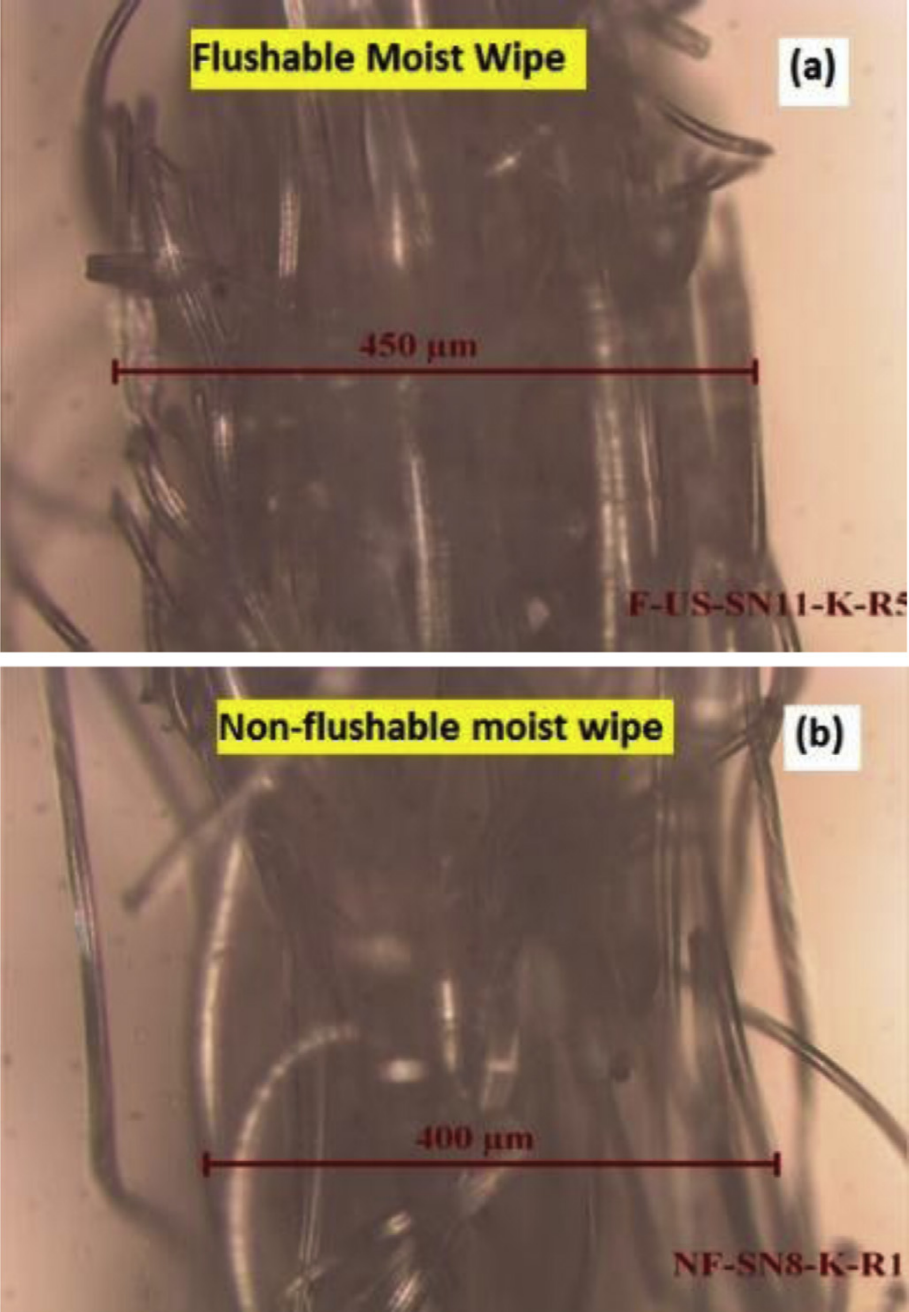
Pictures of cross-sections of moist flushable (panel a), and moist non-flushable wipes (panel b) under a light microscope.

For sheet thicknesses, Fig. 1 depicts typical cross-sections of moist flushable, and moist non-flushable wipes under the light microscope. As illustrated, the cross-section of a wipe appears non-uniform with fibers in slight disarray; therefore, we took five thickness measurements as minimum, maximum, and three other representative readings along the cross-section of each sheet. The arithmetic average of the five measurements was reported as the average sheet thickness.

We present below five thickness measurements and their arithmetic average that is reported as the sheet thickness of sample no.1 in Table 1.

**Table:** 

Thickness measurement-1 (maximum) (μm)	Thickness measurement-2 (minimum) (μm)	Thickness measurement-3 (representative) (μm)	Thickness measurement-4 (representative) (μm)	Thickness measurement-5 (representative) (μm)
390	295	333	322	340
Arithmetic average of five measurements (μm)			(390 + 295+333 + 322+340)/5 = 336	
Reported average thickness of sample no.1 (μm)			336	

By using the measured quantities and the equations below, we estimated other physical properties including sheet volume, basis weight, and specific volume as follows

Sheet volume = surface area x sheet thickness.

Basis weight = Sheet mass/surface area.

Specific volume = Sheet volume/sheet mass.

Accordingly, the computed properties of sample no. 1 of Table 1 are

Sheet volume = 340 (cm^2^) x 336 (μm) x (1 cm/10000 μm) = 11.4 cm^3^,

Basis weight = 6.0 g / 340 cm^2^ x (10000 cm^2^ / 1 m^2^) = 176 g/m^2^,

Specific volume = 11.4 cm^3^ x (1 dm^3^ / 1000 cm^3^) / (6.0 g) x (1000 g /1 kg) = 1.9 dm^3^/kg.

### 1.2 Identification of fiber types through fiber staining

Table 7 presents characteristics of two independent toilet paper (TP) samples that were selected for application of fiber staining methods. Table 8, Table 9, Table 10 present fiber compositions of representative samples. The tables include sample IDs, staining methods, expected colors of fibers, physical description of fibers, and the actual appearance of fibers under a light microscope. Specifically, Table 8 depicts plant fibers of the two independent TP samples, while Table 9 illustrates fibers of two independent wipe samples (non-flushable, and flushable) that are composed of only regenerated cellulose (RC) fibers. Physical characteristics of these samples, NF-SN-11 (non-flushable) and NA-SN-10 (flushable), are available in Table 1, Table 3 of this article, respectively. Table 10 demonstrates fibers of a flushable wipe sample that is composed of plant-based, and RC fibers. Physical characteristics of this sample, EU-SN-5, are available in Table 5 of this document.

**Table 7:** Characteristics of the two toilet paper samples used for fiber analysis in this study.

Parameter	European TP Sample (TP-EU-SN-1)	North American TP Sample (TP-NA-SN-5)
Sheet mass (mg)	622	444
Basis weight (g/m^2^)	50	41
Sheet thickness (μm)	175	130
Sheet volume (cm^3^)	2.2	1.4
Specific volume (L/kg)	3.5	3.3
F_max_ (dry-state) (N)	5.4	2.8
Manufacturer	Global	Global

**Table 8:** Microscopic images of stained plant fibers of two independent TP samples. Fiber appearances match with physical descriptions and expected colors of plant-based fibers as indicated by each staining procedure. Sample IDs indicate TP: Toilet paper, NA: North America, EU: Europe, and SN: Sample Number.

Sample ID	Staining Method	Fiber Description & Expected Fiber Color	Fiber Appearance

TP-EU-SN-1	Herzberg	Non-uniform shape with rough side-edges and pointed-ends. Darkish-bluish violet	
	Dupont	Non-uniform shape with rough side-edges and pointed-ends. Green & yellow	
TP-NA-SN-5	Herzberg	Non-uniform shape with rough side-edges and pointed-ends. Darkish-bluish violet	
	Dupont	Non-uniform shape with rough side-edges and pointed-ends. Green & yellow

**Table 9:** Microscopic images of RC fibers after staining. Fiber appearances match with physical descriptions and expected colors of RC fibers as indicated by each staining procedure. Absence of any other fiber type confirms that the wipe sample is composed of RC fibers by 100%. Sample IDs indicate FL: Flushable, NF: Non-flushable, NA: North America, and SN: Sample Number.

Sample ID	Staining Method	Fiber Description & Expected Fiber Color	Fiber Appearance
NF-SN-11	Herzberg	Long and uniform fibers with smooth side-edges. Darkish-bluish violet	
	Dupont	Long and uniform fibers with smooth side-edges. Greenish-blue	
FL-EU-SN-14	Herzberg	Long and uniform fibers with smooth side-edges. Darkish-bluish violet	
	Dupont	Long and uniform fibers with smooth side-edges. Greenish-blue

**Table 10:** Microscopic images of plant-based, and RC fibers after staining. Fiber appearances match with physical descriptions and expected colors of both plant-based, and RC fibers as indicated by each staining procedure. Sample IDs indicate FL: Flushable, EU: Europe, and SN: Sample Number.

Sample ID	Staining Method	Fiber type	Fiber Description & Expected Fiber Color	Fiber Appearance
FL-EU-SN-5	Herzberg	RC fiber	Long and uniform shape with smooth side-edges. Darkish-bluish violet	
		Plant fiber	Non-uniform shape with rough side-edges and pointed-ends. Darkish-bluish violet	
	Dupont	RC fiber	Long and uniform fibers with smooth side-edges. Greenish-blue	
		Plant fiber	Non-uniform shape with rough side-edges and pointed-ends. Green & yellow	

### 1.3 Tensile properties

Table 11, Table 12, Table 13, Table 14, Table 15, Table 16, Table 17, Table 18 present tensile properties of non-flushable wipes, flushable wipes, and TPs. Essential statistics of the data are available in the last two rows of relevant tables. Our associated article provides interpretations and discussions of the data for further consideration [1]. The data include raw, and analyzed quantities as follows: 1- A tensile property, such as the maximum amount of force (F_max_) that is needed to break a sample, was measured and reported as the average of seven readings for each sample, 2- A tensile property, such as tensile strength, was computed by dividing the measured F_max_ value with width of a specimen. For convenience, we use sample no.1 in Table 11 as an example case to demonstrate step-by-step how we obtained tensile properties of a sample. Accordingly, we used the tensile instrument and measured the F_max_, and the elongation-at-break values for each of seven specimens of a sample. We reported the arithmetic average of seven readings as the measured properties as shown below for sample no.1 in Table 11.

**Table:** 

Specimen no. of Sample 1.	F_max_ (N)	Elongation at Break (% of a specimen length = 100 mm)
1–1	26.03	32.33
1–1	28.70	37.00
1–3	28.05	32.88
1–4	26.54	30.5
1–5	28.80	32.63
1–6	25.64	33.29
1–7	23.63	31.88
Arithmetic average	26.77	32.93
Reported value	27	33

**Table 11:** Tensile properties of dry non-flushable wipe samples from around the world. Tensile properties were quantified by using sheets that were dried at 40 °C for 24 h. Sample IDs indicate NF: Non-flushable wipe, and SN: Sample Number.

No.	I.D.	F_max_ (N)	Tensile Strength (N/m)	Basis weight^(^+) (g/m^2^)	Tensile Index (Nm/g)	Breaking Length (m)	Elongation at Break (%)
1	NF-SN-1	27	1800	52	34.6	3529	33
2	NF-SN-2	4	267	55	4.8	494	8.7
3	NF-SN-3	28	1867	59	31.6	3226	36.4
4	NF-SN-4	12	800	48	16.8	1699	22
5	NF-SN-5	4.6	307	47	6.5	665	19.4
6	NF-SN-6	14.5	967	44	21.9	2240	36
7	NF-SN-7	20	1333	40	33.1	3399	35.7
8	NF-SN-8	15	1000	38	26.1	2683	38.8
9	NF-SN-9	9	600	43	14.0	1423	29
10	NF-SN-10	4	267	58	4.6	469	7.4
11	NF-SN-11	30	2000	59	34.1	3457	23.8
12	NF-SN-12	22	1467	55	26.7	2719	21.9
13	NF-SN-13	9.4	627	51	12.3	1253	12.3
14	NF-SN-14	20	1333	46	28.7	2956	13.5
15	NF-SN-15	14.3	953	52	18.4	1869	33.6
16	NF-SN-16	7	467	77	6.1	618	3.5
	**Average**	**15**	**1003**	**51**	**20.0**	**2044**	**23.4**
	**Range**	**4**–**30**	**267**–**2000**	**38**–**77**	**4.6**–**34.6**	**471**–**3530**	**3.5**–**38.8**
	***s***∗	**9**	**578**	**8**	**11**	**1130**	**12**
	**ε**∗	**2**	**144**	**2**	**3**	**282**	**3**

∗ “*s*” means standard deviation, and “ε” means standard error.

+ Basis weight values were obtained from Table 2 of this article.

**Table 12:** Wet tensile properties of non-flushable wipe samples from around the world. Tensile properties were quantified by using wet sheets of the samples. Sample IDs indicate NF: Non-flushable wipe, and SN: Sample Number.

No.	I.D.	F_max_ (N)	Tensile Strength (N/m)	Basis weight^(^+) (g/m^2^)	Tensile Index (Nm/g)	Breaking Length (m)	Elongation at Break (%)
1	NF-SN-1	28	1867	175	10.7	1088	33
2	NF-SN-2	3	200	215	0.9	95	62
3	NF-SN-3	26.8	1787	207	8.6	880	43
4	NF-SN-4	19.3	1287	137	9.4	958	22.7
5	NF-SN-5	25	1667	166	10.0	1024	30.1
6	NF-SN-6	20.6	1373	124	11.1	1129	38.7
7	NF-SN-7	25.1	1673	108	15.5	1580	38.2
8	NF-SN-8	14.2	947	95	10.0	1016	36.6
9	NF-SN-9	13.1	873	134	6.5	665	25.9
10	NF-SN-10	2.2	147	168	0.9	89	15.6
11	NF-SN-11	18.4	1227	196	6.3	638	23.3
12	NF-SN-12	15.4	1027	191	5.4	548	20.7
13	NF-SN-13	6.8	453	232	2.0	199	15
14	NF-SN-14	10.3	687	205	3.3	342	12.9
15	NF-SN-15	15.3	1020	94	10.9	1106	34
16	NF-SN-16	2.1	140	152	0.9	94	3.4
	**Average**	**15**	**1023**	**162**	**7**	**715**	**28**
	**Range**	**2.1**–**28**	**140**–**1867**	**94**–**232**	**0.9**–**15.5**	**89**–**1580**	**3.4**–**62**
	***s***∗	**9**	**580**	**44**	**4**	**453**	**14**
	**ε**∗	**2**	**145**	**11**	**1**	**113**	**4**

∗ “*s*” means standard deviation, and “ε” means standard error.

+ Basis weight values were obtained from Table 1 of this article.

**Table 13:** Tensile properties of dry flushable wipe samples from North America. Tensile properties were quantified by using sheets that were dried at 40 °C for 24 h. Sample IDs indicate NA: North America (flushable), and SN: Sample Number.

No.	I.D.	F_max_ (N)	Tensile Strength (N/m)	Basis weight^(^+) (g/m^2^)	Tensile Index (Nm/g)	Breaking Length (m)	Elongation at Break (%)
1	NA-SN-1	5.5	367	62	5.9	606	4.6
2	NA-SN-2	5.4	360	64	5.6	578	5.1
3	NA-SN-3	6.2	413	80	5.2	529	10.5
4	NA-SN-4	6.1	407	81	5.0	512	11.4
5	NA-SN-5	6	400	79	5.1	519	11.1
6	NA-SN-6	6.6	440	74	5.9	609	18.5
7	NA-SN-7	4.2	280	59	4.7	481	8.4
8	NA-SN-8	5.1	340	67	5.1	515	4.7
9	NA-SN-9	5.7	380	67	5.7	576	5.6
10^(^β^)^	NA-SN-10	17.8	1187	70	17.0	1724	21.6
11	NA-SN-11	2.8	187	53	3.5	358	7.8
12	NA-SN-12	6	400	79	5.1	517	10.6
13	NA-SN-13	3.5	233	61	3.8	390	11
14	NA-SN-14	7	467	55	8.5	863	12
15	NA-SN-15	7.2	480	58	8.3	845	9.2
16^(^β^)^	NA-SN-16	37.4	2493	51	48.9	4954	30
	**Average**	**5.5**	**368**	**66**	**5.5**	**564**	**9.3**
	**Range**	**2.8**–**37.4**	**187**–**2493**	**51**–**81**	**3.5**–**48.9**	**358**–**4954**	**4.6**–**30**
	***s***∗	**1.3**	**85**	**10**	**1.4**	**142**	**3.8**
	**ε**∗	**0.3**	**23**	**2**	**0.4**	**38**	**1.0**

∗ “*s*” means standard deviation, and “ε” means standard error.

+ Basis weight values were obtained from Table 3 of this article.

β Samples 10 and 16 were excluded from statistical analysis due to their oddly high F_max_ values.

**Table 14:** Wet tensile properties of flushable wipe samples from North America. Tensile properties were quantified by using wet sheets of the samples. Sample IDs indicate NA: North America (flushable), and SN: Sample Number.

No.	I.D.	F_max_ (N)	Tensile Strength (N/m)	Basis weight^(^+) (g/m^2^)	Tensile Index (Nm/g)	Breaking Length (m)	Elongation at Break (%)
1	NA-SN-1	3.5	233	210	1.1	113	16.9
2	NA-SN-2	3.4	227	220	1.0	105	16.3
3	NA-SN-3	1.5	100	202	0.5	51	12.8
4	NA-SN-4	1.4	93	206	0.5	46	15.4
5	NA-SN-5	1.5	100	218	0.5	47	14.7
6	NA-SN-6	1.4	93	194	0.5	49	13.8
7	NA-SN-7	1.5	100	170	0.6	60	14
8	NA-SN-8	2.6	173	209	0.8	85	14.2
9	NA-SN-9	2.8	187	212	0.9	90	12.7
10^(^β^)^	NA-SN-10	18.1	1207	311	3.9	395	26.1
11	NA-SN-11	2.2	147	171	0.9	88	17
12	NA-SN-12	1.8	120	203	0.6	60	13.4
13	NA-SN-13	3.1	207	216	1.0	98	21
14	NA-SN-14	2.9	193	164	1.2	120	15
15	NA-SN-15	2.8	187	186	1.0	102	15.4
16^(^β^)^	NA-SN-16	40	2667	140	19.1	1949	30
	**Average**	**2.3**	**154**	**202**	**0.8**	**80**	**15.2**
	**Range**	**1.4**–**40**	**93**–**2667**	**140**–**311**	**0.5**–**18.1**	**46**–**1949**	**12.7**–**30**
	***s***∗	**0.8**	**52**	**37**	**0.26**	**27**	**2.2**
	**ε**∗	**0.2**	**14**	**9**	**0.07**	**7.1**	**0.6**

∗ “*s*” means standard deviation, and “ε” means standard error.

+ Basis weight values were obtained from Table 3 of this article.

β Samples 10 and 16 were excluded from statistical analysis due to their oddly high F_max_ values.

**Table 15:** Tensile properties of dry flushable wipe samples from European, and from Far Eastern countries. Tensile properties were quantified by using sheets that were dried at 40 °C for 24 h. Sample IDs indicate EU: Europe (flushable), FE: Far East (flushable), and SN: Sample Number.

No.	I.D.	F_max_ (N)	Tensile Strength (N/m)	Basis weight^(^+) (g/m^2^)	Tensile Index (Nm/g)	Breaking Length (m)	Elongation at Break (%)
1	EU-SN-1	6	400	59	6.8	691	10
2	EU-SN-2	5.8	387	55	7.0	722	10.2
3	EU-SN-3	4.2	280	63	4.4	454	14
4	EU-SN-4	8.1	540	65	8.3	852	9
5	EU-SN-5	6	400	65	6.2	624	9
6	EU-SN-6	5.9	393	52	7.6	775	8.8
7	EU-SN-7	5.9	393	48	8.2	844	9
8	EU-SN-8	6	400	63	6.3	650	10
9	EU-SN-9	6	400	68	5.9	604	10
10	EU-SN-10	5.4	360	54	6.7	684	14
11	EU-SN-11	5.2	347	69	5.0	516	8
12	EU-SN-12	3.9	260	60	4.3	439	14
13	EU-SN-13	6.5	433	59	7.3	744	14
14^(^β^)^	EU-SN-14	17.4	993	52	19.1	1946	24
15	EU-SN-15	5.5	367	56	6.6	664	5
16	EU-SN-16	5.3	353	60	5.9	605	9.2
17	EU-SN-17	2.6	173	64	2.7	276	5
18	EU-SN-18	6.4	427	67	6.4	653	12
19	FE-SN-1	3.4	227	42	5.4	691	17
20	FE-SN-2	4.1	273	39	7.0	722	16
21	FE-SN-5	3.4	227	44	5.2	454	8.1
22	FE-SN-6	3.5	233	53	4.4	852	8.3
23^(^β^)^	FE-SN-8	13	500	53	9.4	624	7.4
	**Average**	**6.0**	**381**	**57**	**6.8**	**700**	**11.1**
	**Range**	**2.6**–**17.4**	**173**–**993**	**39**–**69**	**2.7**–**19.1**	**276**–**1946**	**5**–**24**
	***s***∗	**2.1**	**93**	**8**	**1.5**	**145**	**3**
	**ε**∗	**0.45**	**20**	**2**	**0.6**	**31**	**1**

∗ “*s*” means standard deviation, and “ε” means standard error.

+ Basis weight values were obtained from Table 6 of this article.

β Sample 14 was excluded from statistical analysis due to its unusually high F_max_ value. A few samples from Far Eastern Countries were omitted from this list due to inconsistent readings.

**Table 16:** Wet tensile properties of flushable wipe samples from European, and from Far Eastern countries. Tensile properties were quantified by using wet sheets of the samples. Sample IDs indicate EU: Europe (flushable), FE: Far East (flushable), and SN: Sample No.

No.	I.D.	F_max_ (N)	Tensile Strength (N/m)	Basis weight^(^+) (g/m^2^)	Tensile Index (Nm/g)	Breaking Length (m)	Elongation at Break (%)
1	EU-SN-1	2	133	168	0.8	81	10.3
2	EU-SN-2	2	133	160	0.8	85	10
3	EU-SN-3	2.4	160	222	0.7	73	16
4	EU-SN-4	3.3	220	196	1.1	114	16
5	EU-SN-5	2	133	201	0.7	68	14
6	EU-SN-6	2.3	153	141	1.1	111	11
7	EU-SN-7	2.4	160	117	1.4	140	11
8	EU-SN-8	2	133	195	0.7	69	13
9	EU-SN-9	2	133	195	0.7	70	14
10	EU-SN-10	2.1	140	177	0.8	81	12
11	EU-SN-11	3.5	233	227	1.0	105	19
12	EU-SN-12	1.7	113	167	0.7	69	10
13	EU-SN-13	2.5	167	159	1.1	107	15
14^(^β^)^	EU-SN-14	15.5	1240	178	7.0	711	19
15	EU-SN-15	3.5	233	168	1.4	141	16
16	EU-SN-16	2.5	167	233	0.7	75	16.7
17	EU-SN-17	2.3	153	234	0.7	67	16.9
18	EU-SN-18	3.6	240	244	1.0	100	17
19	FE-SN-1	2.2	147	127	1.8	182	17
20	FE-SN-2	2.4	160	111	2.5	251	16
21	FE-SN-5	1.5	100	159	1.4	146	12
22	FE-SN-6	1.4	93	152	1.5	156	6.9
23^(^β^)^	FE-SN-8	1.3	87	136	3.7	375	0.6
	**Average**	**2.9**	**201**	**177**	**1.4**	**147**	**13.5**
	**Range**	**1.3**–**15.5**	**87**–**1240**	**111**–**244**	**0.7**–**7**	**67**–**711**	**0.6**–**19**
	***s***∗	**0.65**	**44**	**39**	**0.7**	**73**	**4.2**
	**ε**∗	**0.14**	**9**	**8**	**0.15**	**15**	**0.9**

∗ “*s*” means standard deviation, and “ε” means standard error.

+ Basis weight values were obtained from Table 5 of this article.

β Sample 14 was excluded from statistical analysis due to its unusually high F_max_ value. A few samples from Far Eastern Countries were omitted from this list due to inconsistent readings.

**Table 17:** Tensile properties of toilet paper samples. Tensile properties were quantified by using sheets that were dried at 40 °C for 24 h. The values are listed in the order of F_max_ values as the highest one on top.

Sample No.	F_max_ (N)	Tensile Strength (N/m)	Basis weight (g/m^2^)	Tensile Index (Nm/g)	Breaking Length (m)	Elongation at Break (%)
1	5.4	358	50	7.2	730	16.8
2	4.6	304	49	6.2	633	17.8
3	4.5	300	57	6.1	537	13.4
4	4.1	274	39	7.0	717	18.3
5	2.8	185	41	4.5	464	10.5
6	2.5	167	44	3.3	617	12.1
7	2.5	167	34	3.7	431	11.8
8	2.4	160	17	9.8	480	5.8
9	2.2	160	28	5.7	340	7.5
10	1.9	127	39	3.3	380	7.5
11	1.6	107	26	4.1	411	3.7
**Average**	**3.1**	**210**	**38.5**	**5.5**	**522**	**11.3**
**Range**	**1.6**–**5.4**	**107**–**358**	**17**–**57**	**3.3**–**9.8**	**340**–**730**	**3.7**–**18.3**
***s***∗	**1.3**	**84**	**12**	**2**	**135**	**5**
**ε**∗	**0.4**	**25**	**4**	**0.6**	**41**	**1.5**

∗ “*s*” means standard deviation, and “ε” means standard error.

**Table 18:** Tensile properties of toilet paper samples in their wet states. The samples are listed in the same order as in Table 17.

Sample No.	F_max_ (N)	Tensile Strength (N/m)	Basis weight (g/m^2^)	Tensile Index (Nm/g)	Breaking Length (m)	Elongation at Break (%)
1	0.28	18.7	50	0.37	38	4
2	0.27	18	49	0.37	37	7
3	0.5	33	57	0.58	60	8
4	0.4	26.7	39	0.68	70	6
5	0.28	18.4	41	0.45	46	6
6	0.06	4	28	0.14	15	2
7	0.13	8.7	39	0.22	23	2
8	0.33	22.1	34	0.65	66	7
9	0.38	25	44	0.57	58	8
10	0.18	12.2	34	0.36	37	3
11	0.025	1.7	26	0.1	7	∼1
**Average**	**0.26**	**17.1**	**40**	**0.4**	**41.5**	**4.9**
**Range**	**0.025–0.5**	**1.7**–**33**	**26**–**57**	**0.1–0.68**	**7**–**70**	**∼1**–**8**
***s***∗	**0.15**	**10**	**9.5**	**0.2**	**21**	**2.4**
**ε**∗	**0.04**	**3**	**3**	**0.06**	**6**	**0.7**

∗ “*s*” means standard deviation, and “ε” means standard error.

By using the measured quantities and the equations below, we estimated tensile strength, tensile index, and breaking length of a sample as follows

Tensile Strength = F_max_/width of a specimen

The F_max_ value for sample no. 1 (NF-SN-1) was reported as 27 N in Table 11, and width of a specimen was 15 mm = 0.015 m. Hence, tensile strength is 27/0.015 = 1800 N/m as shown in the fourth column of Table 11. Likewise, tensile index is

Tensile Index = Tensile Strength/Basis weight

Basis weight of sample no. 1 (NF-SN-1) in Table 11 was obtained as 52 g/m^2^ from Table 2 of this article. Accordingly, Tensile Index is 1800 (N/m)/52 (g/m^2^) = 34.6 Nm/g for the sample (6th column of Table 11). Finally, breaking length is

Breaking Length = Tensile Index (Nm/g)/Gravitational Acceleration

Breaking Length of a sample is the length at which the sample will break due to its own weight. Accordingly, breaking length for sample no. 1 (NF-SN-1) in Table 11 is 34.6 (Nm/g) x 1000 (g/kg)/9.807 (m/s^2^) = 3529 m.

## 2 Experimental design, materials, and methods

### 2.1 Microscopic thickness measurements

**Fig. 2:**
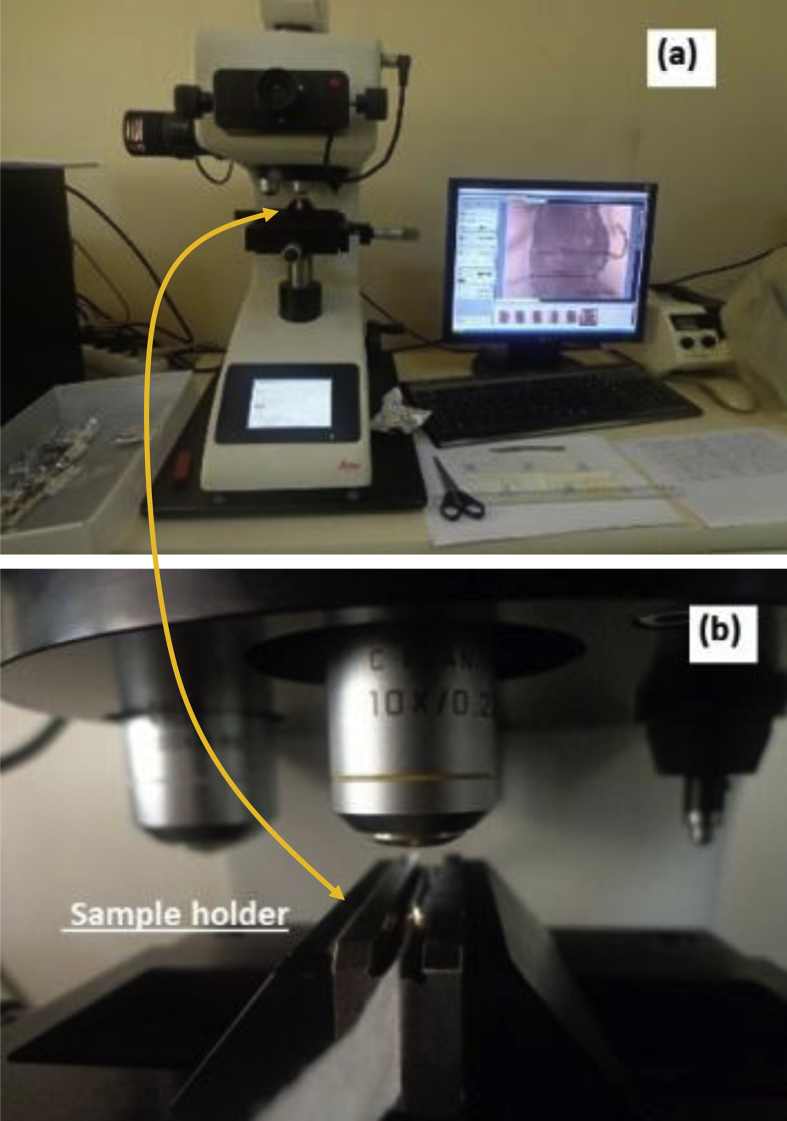
Sheet thickness measurements of wipes by using a light microscope (Leica VMHT MOT, Wetzlar, Germany). (a) General view of the microscope and its image processing software running on a personal computer. Cross-section of the wipe sample is shown on computer screen, where horizontal red lines indicate sheet thicknesses measured at various points along the cross-section. (b) Typical view of a wipe sample fastened vertically to sample holder.

For thickness measurements, we used a light microscope, (Leica VMHT MOT, Leica Microsystems GmbH, Wetzlar, Germany) at 100× magnification. This microscope can measure thicknesses as low as 50 μm at increments of 1 μm. Fig. 2 illustrates with photographs how sheet thicknesses were quantified for each wipe or TP sample.

### 2.2 Tensile properties of nonwoven wipes and toilet papers

**Fig. 3:**
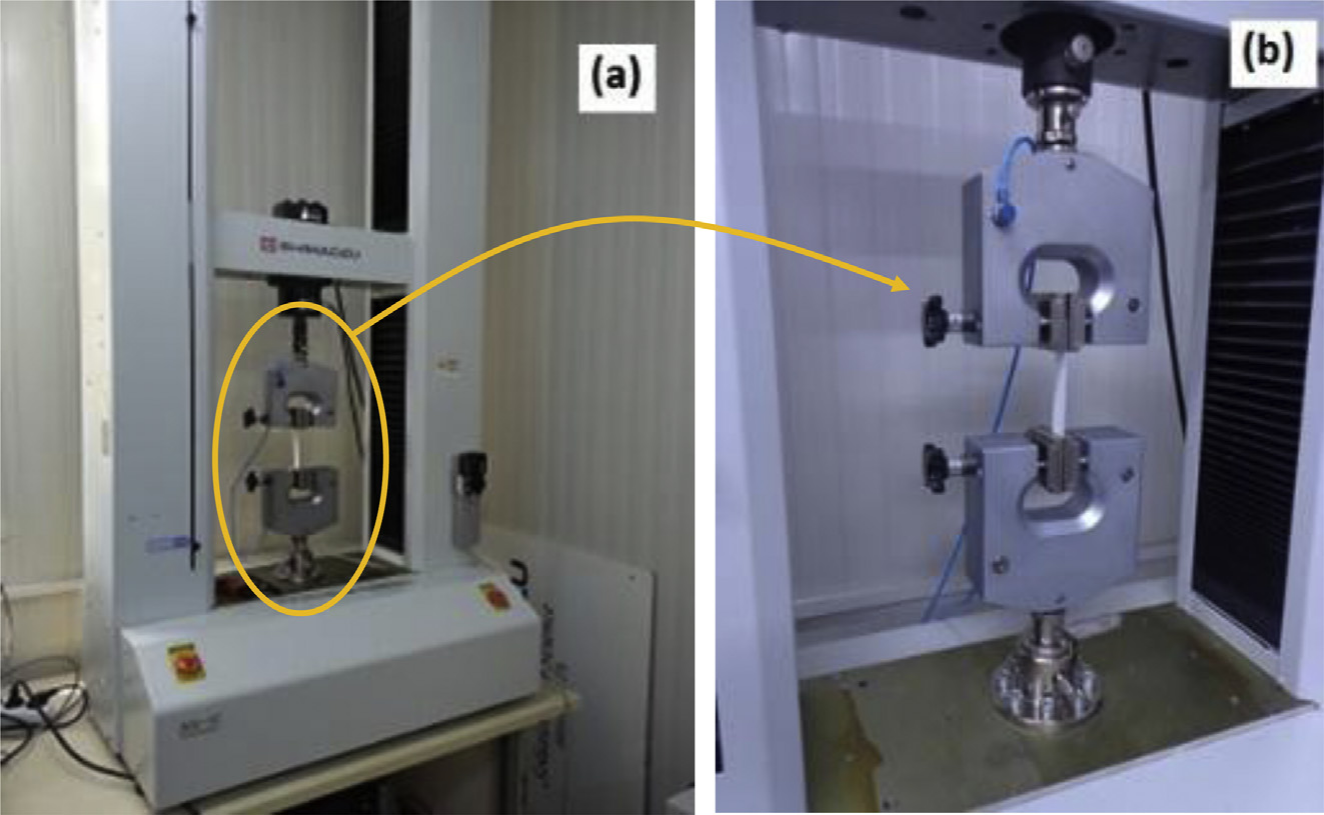
Measurement of tensile properties of nonwoven wipes and TPs by using universal tensile testing machine (Schimadzu AG-IC, Tokyo, Japan). (a) General view of the tensile testing machine, (b) Close-up view of the jaws where a strip was fastened for testing.

Tensile properties of dry samples were measured according to standard method of ISO 12625 - Part 4: Tissue paper and tissue products – Determination of (dry) tensile strength, and stretch at break by using tensile apparatus operating with constant rate of elongation. We used a universal tensile testing machine (Schimadzu AG-IC, Tokyo, Japan) that was controlled by Trapezium X Materials Testing Software. The tensile instrument has two jaws with clamps that hold a strip of a sample in place during testing. The bottom jaw is fixed, while the upper jaw elongates a strip during a test run. For calibration of the instrument, we tested standard printing paper, toilet papers, and standard cardboard, for which tensile properties were available. Then, we prepared a single strip of a wipe (or TP) as 15 mm in width and >100 mm in length, and placed exactly 100 mm between jaws of the tensile machine. During testing, the upper jaw pulled a strip upwards, while the Trapezium X software controlled rates of loading, and separation of jaws. Force was applied at increments of 0.1 N, while the jaws were separated at a constant rate of 25 mm/min, and each strip broke between 15 and 30 seconds. Collectively, these steps confirmed constant rate of elongation for each strip as required by the ISO method. In accordance, we took 5 to 7 measurements for each specimen, and we rejected any strip that broke near jaws. In addition, we tested samples in their machine directions (MD), and in their cross directions (CD), and we report the results in MD to be succint. Fig. 3 shows the instrument, and a close-up view of a strip fastened to the instrument.

**Fig. 4:**
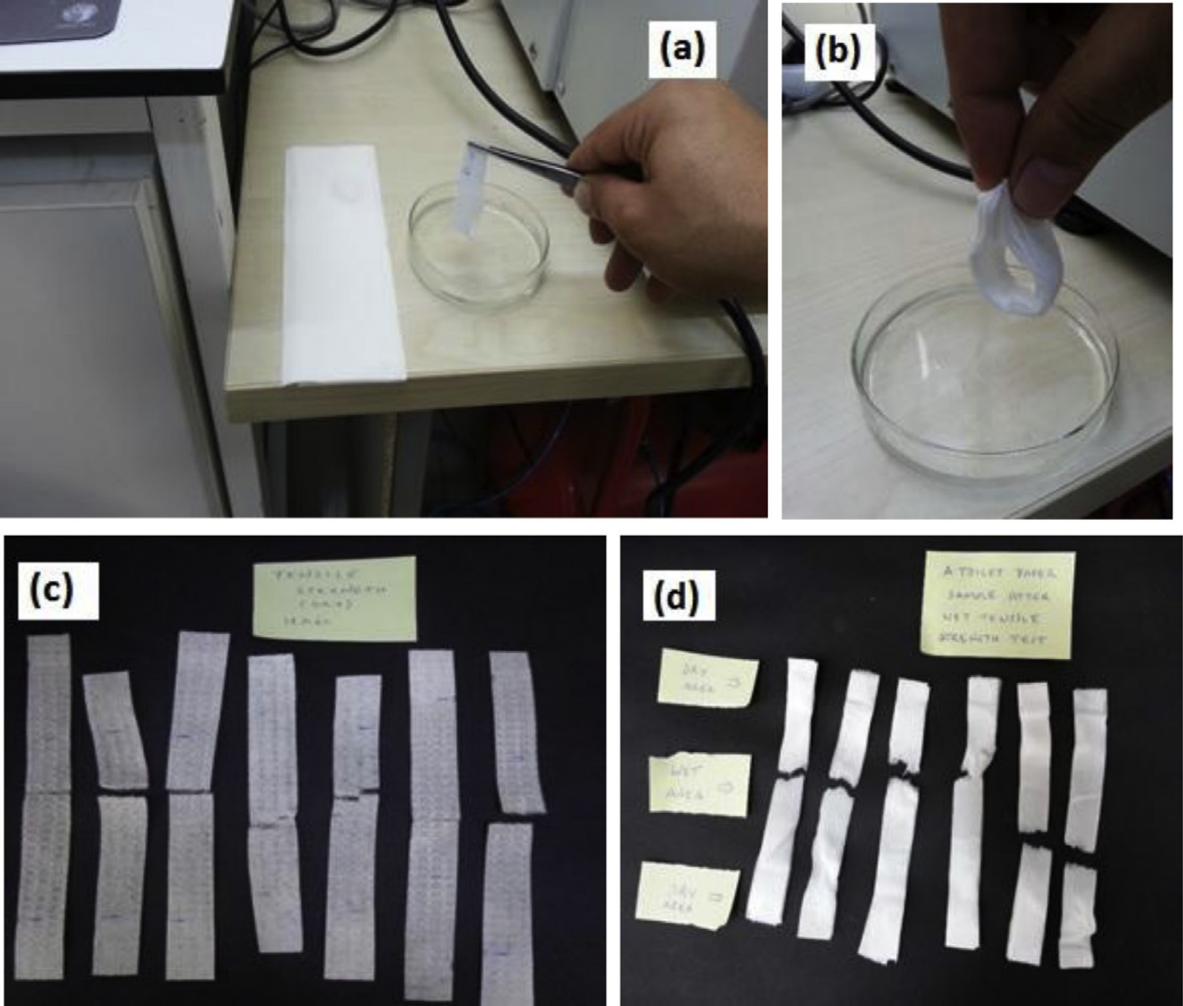
Quantification of tensile properties of nonwoven wipes and TPs in wet states. Panel (a) shows that a single strip of moist wipe was rolled around and dipped into deionized water for saturation. Then, it was fastened to the jaws of the tensile instrument for testing as shown in Fig. 3. Panel (b) shows 6 strips of a TP that are rolled around and dipped into deionized water for saturation. By this approach, only the central part of TP strips were wetted, while the edges remained dry so that strips were fastened to the tensile machine properly. Panel (c) shows a typical view of wipe specimens after test runs. Panel (d) shows typical view of TP specimens after test runs. Each specimen in panel (d) is composed of 6 strips of a TP sample.

Tensile properties of wet samples were measured according to standard method of ISO 3871: Determination of tensile properties after immersion in water. For very absorbent papers such as TPs, the standard method indicates that only the central part of the test specimen should be wetted. In accordance with this guideline, we rolled around a strip like a circle, and dipped its central area into deionized water for 5 seconds for saturation with water. Then, we fastened the strip to the machine and started the tensile test immediately. Fig. 4 depicts the procedure, and pictures of strips after test runs. For TPs, we placed 3 or 6 strips on top of each other, wetted their central part, and conducted our testing. Using 3 or 6 strips of a TP allowed us to obtain realiable and repeatable readings, e.g., F_max_ readings were well above the instrument's minimum reading limit of 0.1 N. Then, we divided the measured F_max_ by the number of strips to estimate the F_max_ value for a single sheet. Standard deviation (*s*) and standard error (ε) of F_max_ values for wet TPs were 0.15, and 0.04, respectively, while the mean (average) F_max-wet_ value was 0.26 N for TPs (Table 17, Table 18). Accordingly, standard deviation was (0.15/0.26)×100 = 58%, and standard error was (0.04 N/0.26 N) = 0.15 × 100 = 15% of the average F_max_ value of 0.26 N. The relatively high *s* and ε values are attributable to material compositions of TP samples. Briefly, fiber types (e.g., hardwood, softwood), their mixing ratios, fiber strength, fiber dimensions, as well as, types and amounts of binders and wet strength resins that are used to form a final product collectively influence that product's strength in its dry, and in its wet states. Hence, F_max_ quantities varied considerably for TPs in their wet states as indicated by the *s* and ε values of our measurements.
